# Uptake of Front-of-Package Nutrition Labeling Scheme after 5 Years of Adoption in Thailand: An Analysis of New Launched Pre-Packaged Food and Beverages Products

**DOI:** 10.3390/nu15143116

**Published:** 2023-07-12

**Authors:** Hung Nguyen Ngoc, Juntima Photi, Nattapol Tangsuphoom, Wantanee Kriengsinyos

**Affiliations:** 1 Doctor of Philosophy Program in Nutrition, Faculty of Medicine Ramathibodi Hospital and Institute of Nutrition, Mahidol University, Nakhon Pathom 73170, Thailand; nghung9010@gmail.com; 2 Food and Nutrition Academic and Research Cluster, Institute of Nutrition, Mahidol University, Salaya, Phutthamonthon, Nakhon Pathom 73170, Thailand; juntima.pho@mahidol.ac.th (J.P.); nattapol.tng@mahidol.ac.th (N.T.)

**Keywords:** front-of-package (FOP) label, nutrition labeling, food policy, public health nutrition, Thailand

## Abstract

In 2016, Thailand introduced voluntary front-of-pack nutrition labeling, the ‘Thailand Healthier Choice’ logo (THCL), in order to help consumers make informed, healthier food choices in each food category. This study aimed to assess the uptake of the THCL scheme in Thailand after five years of implementation by analyzing a newly launched product database. Data on the nutritional composition and labeling were obtained from the Mintel Global New Products Database between 2017 and 2021. The product’s healthfulness was determined using the Health Star Rating (HSR) algorithm. The numbers and proportions of total, eligible, and labeled products bearing the THCL logo were analyzed and classified by food category, by HSR value, and by manufacturer. After 5 years of implementation, THCL uptake as a proportion of total products and eligible products continues to increase by 3.0% and 10.2% per annum, respectively. The logo has correspondingly appeared on 10.7% and 39.5% of total and eligible products. As a voluntary implementation, 76.1% of products displaying the THCL logo belonged to the ‘non-core’ group, i.e., non-alcoholic beverages and instant foods. This food category (HSR < 3.0) was more likely to register to bear THCL rather than those ‘core’ food scoring an HSR ≥ 3.5, which might reflect economic considerations and benefits. The present analysis also found that only 10% of manufacturers in Thailand launched ‘healthier’ products that display the THCL logo with varied product numbers. To summarize, the initial five-year implementation of the THCL program shows promise, but its adoption remains limited and inconsistent, thereby restricting its influence on public health. Our discoveries highlight the limitations of commercial goodwill in applying THCL voluntarily in Thailand and offer potential suggestions to enhance its adoption in the coming years.

## 1. Introduction

Obesity and its health consequences, i.e., non-communicable diseases (NCDs) such as diabetes, cardiovascular diseases, and cancer, have become significant public health concerns and account for 74% of worldwide deaths [1]. The increase in the consumption of unhealthy diets, particularly in foods with high contents of ‘undesirable’ nutrients, such as energy, fatty acids, sugar, and sodium, associated with changes related to an increasingly sedentary lifestyle, has largely contributed to the salient growth in the number of people affected by obesity [2]. Notwithstanding this factor, it is recognized that obesity has a multidimensional etiopathogenesis, and it is unlikely that a single preventive approach will significantly reduce the risk of disease, but providing information, assistance, and guidance to consumers could be the first potential tool to move toward healthier food choices [3]. As part of evidence-informed measures, the World Health Organization (WHO) recommends front-of-pack nutrition labeling (FOPNL) on packaged foods and beverages in order to promote healthy diets by facilitating consumers’ understanding of the nutritional values of food and helping them make healthier food choices. Additionally, this prominent nutritional policy also aims to improve the availability, affordability, and acceptability of healthier food products by driving the reformulation process in the food industry [4,5].

Consequently, numerous FOPNL schemes, initiated by governments, have been devised and implemented in various countries, adopting different approaches, such as positive or negative, and varying between mandatory and voluntary implementations [6]. In 2016, Thailand voluntarily adopted the conclusive interpretive FOPNL scheme under the ‘Thailand Healthier Choice Nutritional Labeling’ (THCL) logo [7]. The THCL system uses nutrient profiling criteria to classify and highlight healthier options within a food group individually, targeting both ‘desirable’ nutrients (e.g., protein and dietary fiber) and ‘undesirable’ nutrients (e.g., energy, total fat, saturated fatty acid, sugar, and sodium). In order to bear the THCL logo, products should meet the nutritional requirements set under their respective criteria [7].

**Figure 1:**
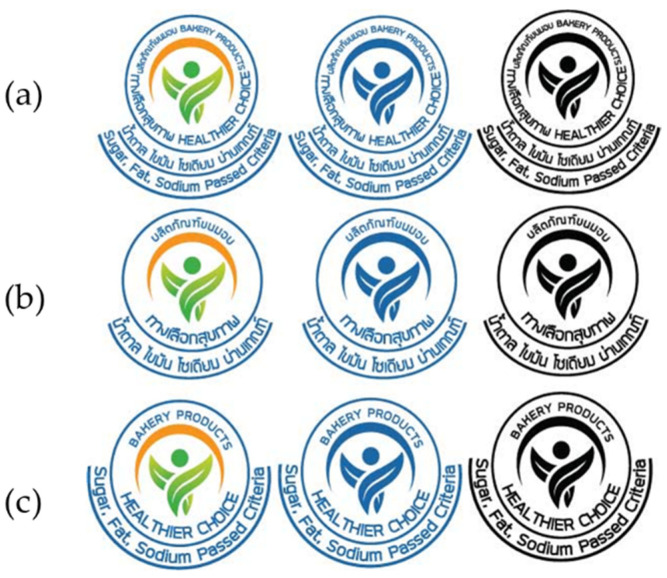
The Thailand Healthier Choice nutrition labeling graphic in different languages. (**a**) English and Thai language; (**b**) Thai language; and (**c**) English language.

Structurally, the THCL system comprises three components: the THCL nutrient criteria set, the THCL graphic (Figure 1), and an accompanying education and communication campaign [8]. The THCL criteria used for each food category are established according to the technical guidance of the sub-committee on the development and promotion of simplified nutrition labeling and are approved by the National Strategic Steering Committee on the linkage between food and nutrition for a better quality of life under the Thai National Food Committee. Since its adoption in 2016 with 3 food groups, the extension of eligible food categories has continuously developed, with 14 food groups to date (Supplementary S1). Nevertheless, the issuance of the THCL logo is restricted for products targeting specific consumer groups or serving specific purposes. This includes exclusions for products such as infant formula, drinking water, mineral water, and other specialized foods, including medical food, dietary supplements, and alcoholic beverages [8].

As a prominent intervention with potential impacts on public health, close monitoring and annual reporting should be methodically implemented. By analyzing the newly launched products in the Thai market during the period between 2017 and 2021, the objective of this investigation is to provide an update on the progress of THCL uptake in Thailand after five years of implementation and provide insight into the feasibility of the implementation and expansion of the THCL scheme in Thailand.

## 2. Materials and Methods

### 2.1. Data Source

The primary analysis of THCL uptake comprised between-year comparisons of serial cross-sectional surveys of newly launched pre-packaged foods in Thai markets during 5 years of implementation. Systematic sampling of all new packaged food and beverage product launches in the Thai market using the Mintel Global New Products Database (GNPD) between 1 January 2017 and 31 December 2021 was conducted. Mintel is an industry resource that indexes all new food and beverage products launched (i.e., food production innovations) in specific markets covered by the company [9]. All new products launched during the specific period were obtained. Detailed information on all products was extracted, including the presence of THCL displayed, GNPD food category and subcategory, release date, product description, barcode, manufacturer, nutritional composition, and ingredient list.

### 2.2. Food Categorization

Based on the availability of the database, in this investigation, we categorized newly launched packaged food and beverage products into 18 main food categories (Supplementary S4). Data transformation was performed in 3 main steps:

Step 1: The data were checked and cleaned based on the product’s name, description, ingredient list, and appropriateness for the main and minor food categories coded in the Mintel GNPD. The Mintel GNPD classifies food and beverage products using a hierarchical system; specifically, it divides foods into major groups (e.g., dairy) and minor categories (white milk, flavored milk, etc.). Originally, the obtained database included 15 major food categories and 45 minor categories (Supplementary S3).

Step 2: Based on the product information, these items and their corresponding GNPD minor food categories were transformed into THCL minor food groups. For example, RTD (ice) tea and tea in the GNPD were transformed and merged into instant tea in the THCL categorization. This investigation analyzed products launched during the time frame from 2017 to 2021, and thus, only 12 THCL food categories and 35 subcategories were included (excluding meat and poultry products, fish, and other aquatic products, which are new food categories added to the THCL criteria at the end of 2021 and 2022) (Supplementary S1). This food categorization was applied to examine and classify whether products are eligible for displaying the THCL logo.

Step 3: For reporting purposes, the 35 THCL food sub-groups were then transformed into 18 main food groups as follows: (1) bakery products; (2) bread; (3) breakfast cereal; (4) dairy products; (5) fats and oils; (6) ice cream; (7) instant foods; (8) instant tea and coffee; (9) malted and chocolate drinks; (10) plant-based milk substitutes; (11) prepared meals; (12) seasoning; (13) small meals; (14) starch- and meat-based snacks; (15) nut- and bean-based snacks; (16) soups; (17) soft drinks; and (18) vegetable and fruit juices (Supplementary S4). These food groups were mainly based on the THCL categorization with the stratification within the non-alcoholic beverage group.

### 2.3. Nutrition Labeling, Nutritional Composition Data, and Included Samples

Data from the Mintel GNPD were extracted into a Microsoft Excel spreadsheet (Redmond, WA, USA) for further analysis. In this database, the presence or absence of THCL logos on products has been routinely determined at data entry since 2017 by examining images of product front-of-package labels. The presence of THCL was not consistently recorded in 2016, as this was the year of THCL’s adoption and was therefore taken as zero for this paper.

Regarding food and nutritional composition data, the information for data entry included the mandatory back-of-package nutrition information panels (NIPs) per serving and per 100 g or mL, including energy (kcal), total fat (g), saturated fat (g), protein (g), total carbohydrate (g), fiber (g), total sugar (g), and sodium (mg). Data on calcium and iron were also inputted, expressed as the percentage of recommended dietary allowance (RDA) and then computed to mg per 100 g or mL. In Thailand, it is not mandatory to report details on fiber, non-concentrated fruits, vegetables, nuts, and legumes (FVNL) (%), and concentrated FVNL (%) on NIPs. Thus, if such details were absent, appropriate values were determined and estimated according to information drawn from the back-of-package ingredient list, Thai generic food composition databases, or similar product comparisons. The fiber and FVNL estimation was implemented as follows:

Step 1: The ingredient lists were scanned and reviewed for (a) fiber-containing ingredients (inulin, psyllium pectin, fructooligosaccharides, etc.) and (b) fruit-, vegetable-, nut-, and legume-containing ingredients.

Step 2a (for fiber estimation): With regard to the minor category that contained a similar range of products (popcorn, dressing, wet soups, etc.), an average value was computed from all products disclosing fiber values (excluding a zero value). This average amount was then designated to fiber products having missing fiber values but listing fiber-containing ingredients in that minor food category. In contrast, in the food sub-group that had a more diverse range of products, products with missing fiber values were matched to similar products (with declared fiber).

Step 2b (for FVNL estimation): Based on the ingredient list, the estimation of concentrated and non-concentrated FVNL was conducted following the recommended decision tree [10]. Concentrated and non-concentrated FVNL were correspondingly allocated as <25% and ≤40% (FVNL is not the first three or two ingredients); ≥25% and >40% (FVNL is the third or second ingredient); ≥67% and >80% (FVNL is the first ingredient, and non-FVNL ingredients do not substantially contribute to the product’s weight); and 100% (FVNL is the sole ingredient contributing to the product’s weight).

**Figure 2:**
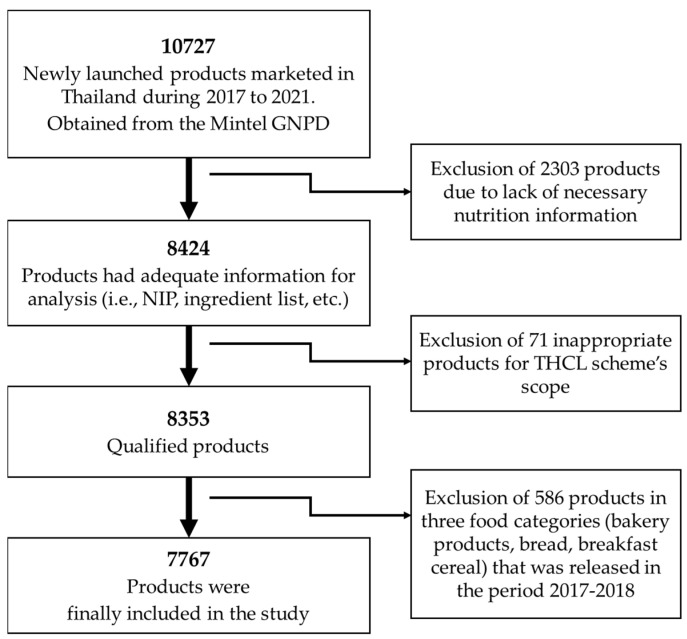
Flowchart of the total number of products included in this analysis.

To ensure the data quality, a second researcher doubled checked the data for any inconsistencies and errors. The data were then cleaned and analyzed by trained nutrition researchers. Duplicate products were identified. For all products, NIP data for the product, as prepared according to manufacturer instructions, were used. If these data were not available, the products were excluded from the analysis (Figure 2). The maximum and minimum nutritional values were checked and compared to package images to ensure accuracy. NIP data for products where data were displayed ‘per serving’ were standardized to per 100 mL or 100 g, depending on the form of the food. Energy values displayed on the packaging, measured in kilocalories, were converted to kilojoules using the conversion factor of 4.184 from the International System of Units [11]. Values listed on the NIP as ‘less than’ were adjusted to their closest whole number for analysis. For example, fiber <1.0 g/100 g was adjusted to 1.0 g/100 g. In order to analyze the uptake of THCL by food manufacturers, we also extracted information on the ultimate company for each product, which was identified by its English trade name.

This investigation covered only pre-packaged food and beverage products. We excluded the following food items, as they were inappropriate for the THCL scheme’s scope: drinking water, vinegar, supplementary sports foods, infant foods and formulas, foods for special medical purposes, vitamins and supplements, etc. (Figure 2). All products were identified using their unique barcodes. Where an item appeared in more than one package size (i.e., 330 mL can or 500 mL bottle of the same beverage), each package size was considered an individual product [12]. This approach’s purpose is to capture all possibilities to label THCL in different product packages that have been updated by manufacturers.

### 2.4. Calculation and Classification of Health Star Rating Values

In this study, the healthfulness of each product was determined using the Health Star Rating (HSR) calculator. This Nutrient Profiling Scoring Criterion (NPSC) algorithm was established by Food Standards Australia New Zealand (FSANZ) and was published previously [13]. The HSR scheme is a voluntary interpretive FOPNL for packaged foods adopted in Australia and New Zealand. The system assigns a value between 0.5 and 5.0 stars, in half-star increments, with a higher number of stars indicating a healthier product in that category. The HSR was calculated in alignment with the methods described in the ‘Guidance for Industry to the Health Star Rating Calculator v7 (March 2023)’ [13]. The HSR scheme divided food and beverages into 6 main groups (i.e., Categories 1, 1D, 2, 2D, 3, and 3D). As to the limitation of eligible food groups for the THCL scheme, only four HSR categories were determined in this analysis, except Categories 3 (oils and spreads) and 3D (cheese and processed cheese). The HSR category was determined by considering factors such as the product name, nutrient composition, and ingredient list. This categorization included non-dairy beverages (Category 1), dairy beverages (Category 1D), dairy foods (Category 2D), and non-dairy foods (Category 2) excluding those found in Category 3 and Category 3D. To calculate the HSR value, the following steps were conducted: (1) assigning baseline points for energy (kJ/100 g or mL), saturated fat (g/100 g or mL), total sugar (g/100 g or mL), and sodium content (mg/100 g or mL); (2) awarding modifying points for FVNL content, protein, and fiber where applicable; (3) calculating an overall score by subtracting modifying points from baseline points, with a lower score reflecting a more nutritious food product; and (4) assigning an HSR according to the overall score using the defined scoring matrix [13].

Although there is yet to be an official benchmark of an HSR score for ‘healthier’ or ‘less healthy’ products, several previous studies compared the correlation between HSR (10-grade scheme) and Nutri-Score (5-grade scheme) in a large food database. Nutri-Score is a summary FOPNL popularly adopted in European countries and identifies the nutritional quality of a product using a 5-color scale, ranging from dark green (aligned with the letter A) to dark red (aligned with the letter E) [14,15,16]. Earlier comparative results revealed that the HSR grades (4.5–5 stars) are compatible with Nutri-Score letter A, 3.5–3 stars are compatible with letter B, and so on [14,15,16].

### 2.5. Statistical Analysis

The outcome of this study is the determination of the percentage uptake of THCL in newly released products marketed in Thailand during 5 years of implementation. To further understand the actual uptake of THCL, the proportion uptake was calculated as a percentage of total products and eligible products and was determined individually for each year. This analysis was conducted for the period between 2017 and 2021. The computation to calculate the uptake is shown in the following equations:

(1) $$Uptake\;per\;total\;product=\frac{Number\;of\;product\;displaying\;THCL}{Total\;newly\;launched\;product}$$

(2) $$Uptake\;per\;eligible\;product=\frac{Number\;of\;product\;displaying\;THCL}{Total\;newly\;launched\;eligible\;product}$$

To forecast the future uptake of the THCL scheme, we performed a thorough analysis of goodness-of-fit using various linear and nonlinear models. This involved evaluating the residuals and coefficient of determination (R-squared) to determine the appropriateness of each model. The estimated linear trend per annum, along with its 95% confidence interval, was calculated to project the THCL uptake trendline from 2019 to 2027. These projections were based on the actual uptake data obtained from the period between 2017 and 2021 [17]. Two projections were created: (1) uptake as a proportion of total products and (2) uptake as a proportion of eligible products. The linear trend equation is y=a+bX , where “a” is the intercept, “b” is the slope of “X” , and “ X” is the period being forecasted. The calculation of “a” and “b” followed this equation:

(3) $$a=\frac{\sum y-b\sum t}{n};b=\frac{n\sum ty-\sum t\sum y}{n\sum t^{2}-(\sum t{)}^{2}}$$

To provide more insights into THCL uptake, the proportions of products were further classified by food category (from the 18 major food categories), by HSR value, and by food manufacturer. Differences in the proportion of uptake were examined for statistical significance using the Chi-square test at *p*-value < 0.05. All statistical analyses, including figures and linear trend generation, were performed using the software GraphPad Prism version 9.5 (GraphPad Software, San Diego, CA, USA).

## 3. Results

### 3.1. THCL Uptake over Time

During the period between 2017 and 2021, a total of 8353 newly launched products marketed in Thailand were qualified in this analysis. In order to reflect the true proportion of THCL uptake among newly released products in food groups whose THCL criteria were latterly introduced (i.e., bakery products, bread, and breakfast cereal), their values for total newly launched and eligible products were counted only in the period between 2019 and 2021. Thus, 7767 products were finally included in this investigation. Among them, 2097 (27.0%) products were eligible for bearing THCL, while 829 (10.7%) products displayed THCL.

The trend of uptake as a proportion of total products during 2017–2021 suggests an approximately linear increase of 3.0% (1.9–4.2%) per annum. If this linear trend is maintained, uptake could reach approximately 32.1% by 2027. On the other hand, the trend of THCL uptake for eligible products was found to be higher, with a linear increase of 10.2% (4.9–15.6%) annually. If this trend is continually upheld by manufacturers, this could allow the uptake of THCL to reach approximately 100% by 2027, which means that all eligible newly launched products would register to bear the THCL logo after 10 years of implementation.

**Figure 3:**
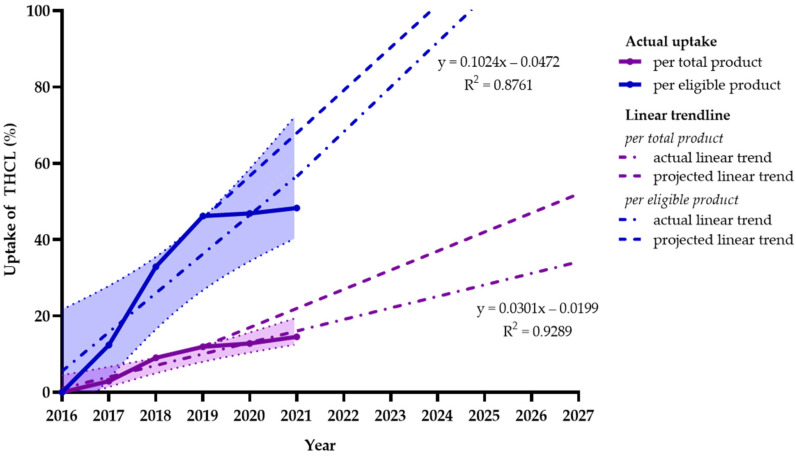
Uptake (%) and its confidence interval of Healthier Choice nutrition labeling on total newly launched products (purple line) and eligible products (blue line) in Thailand from 2017 to 2021 and projection to 2027. The blue and purple bands surrounding the actual uptake lines indicate the confidence intervals. The actual linear trends are represented by the equations (purple dashed dot line) y = 0.0301x − 0.0199, R^2^ = 0.9289 and (blue dashed dot line) y = 0.1024x − 0.0472, R^2^ = 0.8761. Projected linear trends in uptake for total products (purple dashed line) and eligible products (blue dashed line) were estimated based on the uptake in the first two years after adoption.

Figure 3 describes the actual and projected linear trends of the uptake of THCL as a proportion of total products and eligible products from 2017 to 2021 and its projection to 2027. As shown in this illustration, we found negative effects of the COVID-19 pandemic on product launching and the uptake of the THCL scheme. In particular, during the outbreak period between 2019 and 2021, the uptake was found to be modest, with corresponding increases in uptake for total products and eligible products of 1.3% (−2.1–4.7%) and 1.1 (−1.5–3.7%). These figures were lower compared to statistics during the pre-pandemic period, when the average uptake for total and eligible products was reported at 4.2% (2.3–6.1%) and 15.9% (11.2–20.6%), respectively.

### 3.2. THCL Uptake by Food Category

**Figure 4:**
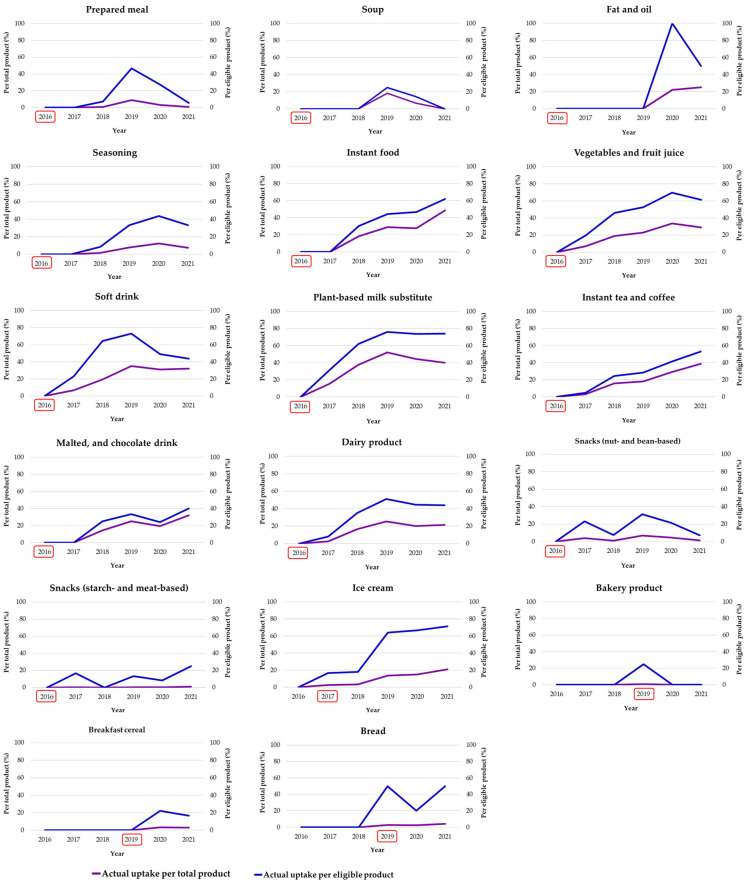
The uptake (%) of Healthier Choice nutrition labeling by food category for total newly launched products (purple line) and eligible products (blue line) in Thailand from 2017 to 2021. Note: The year with the red rectangular box is the first year that the THCL scheme was adopted for that food category; the figure for the uptake of the small meal category was omitted because the uptake was null.

Although the proportion of uptake was different, the trend of THCL uptake for total products and eligible products shared a similar pattern (Figure 4). The figures indicate that the uptake of THCL was neither widespread nor consistent. In non-alcoholic beverages, one of the earliest groups for which THCL criteria were established in 2016, the uptake of THCL began in the year after and gradually increased over time. Soft drinks, plant-based milk substitutes, and vegetable and fruit juices experienced an uptake of 60–80% of eligible products and 40% of total products. In contrast, solid foods (e.g., prepared meals, soups, and seasoning) slowly responded to the THCL scheme. The first uptake was observed 2–3 years after its adoption, with a proportion of 20–40% of eligible products and less than 10% of total products. The fat and oil group (i.e., dressing) also witnessed the first uptake of THCL 4 years after the criteria’s introduction.

Notably, the uptake of THCL for instant foods was interesting: the first uptake was reported 2 years after its adoption, but there was a gradual increase in later years; specifically, the uptake reached approximately 20% in 2018 and jumped to 40% in 2020 and 60% in 2021. The difference in uptake between total products and eligible products was around 10%. In the snack group, also one of the earliest food groups having THCL criteria, the uptake of THCL was found to fluctuate greatly. In particular, the uptake of THCL was reported at 10–20% and 20–30% of eligible products and less than 10% and 5% of total products in starch- and meat-based and nut- and bean-based snacks, respectively.

In 2017, the THCL criteria for ice cream were first introduced and received an instantaneous response from the food industry. The uptake of THCL for both total products and eligible products in this food category was reported at 20% in the first 2 years and jumped to 60% in later years. On the other hand, starchy groups (i.e., bakery products, breakfast cereal, and bread) showed an inconsistent uptake. The uptake for eligible products was recorded at approximately 20–40%, in comparison with only 5% of total products.

Table 1 describes the detailed figures of the uptake of THCL classified by food category. In the top position, beverage and instant food industries had the highest uptake, particularly plant-based milk substitutes (37.7%), soft drinks (25.8%), instant foods (24.1%), instant tea and coffee (23.6%), vegetable and fruit juices (22.0%), and malted and chocolate drinks (17.4%). In contrast, breakfast cereal (2.1%), bakery products (0.3%), and starch- and meat-based snacks (0.3%) had the lowest proportion of uptake. When estimating the annual trends among the food categories, the yearly rate was found to be the highest among instant foods (9.7% per annum). Beverage industries were the runner-up, with a linear increase of 6.5% to 8.6% per annum. Dairy products and ice cream shared moderate uptake trends at approximately 4.8 and 4.4%, respectively. In the last place, snacks, bread, bakery products, and breakfast cereal showed a very slow uptake trend (less than 1%).

Of the 2139 products eligible for THCL logos, plant-based milk substitutes (64.1%) and ice cream (56.8%) had the highest uptake, followed by beverage groups, such as vegetable and fruit juices (51.2%), soft drinks (50.8%), and dairy products (37.6%). On the other hand, breakfast cereal (9.7%), soups (10.2%), bakery products (18.1%), and starch- and meat-based snacks (11.7%) had the lowest uptake.

In this analysis, we observed notable advancements in the nutritional profiles of instant foods (including soups) and non-alcoholic beverage industries following 5 years of THCL implementation. A substantial proportion (>50.0%) of newly launched products in these categories qualified for THCL eligibility, indicating significant improvements in their nutritional attributes. Conversely, bread, bakery products, ready-to-eat meals, and starch- and meat-based snacks had a low proportion of ‘healthier’ products in their portfolios (<10.0%).

### 3.3. THCL Uptake by Healthfulness Level

**Figure 5:**
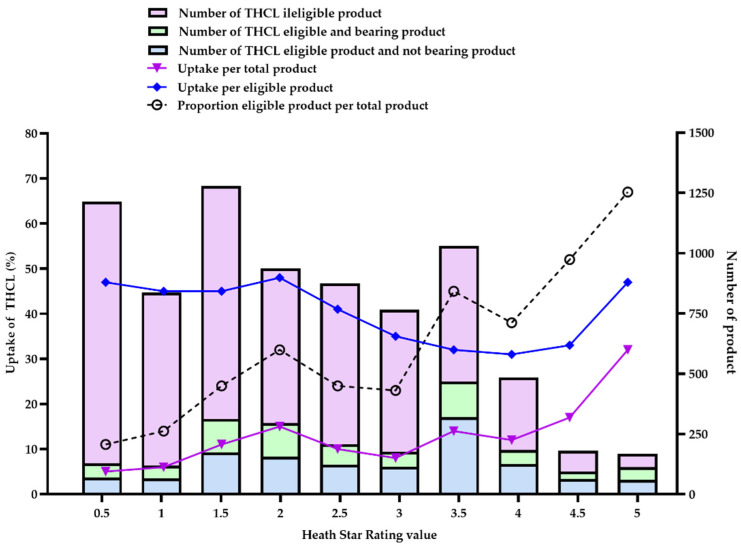
Uptake (%) of the THCL logo across HSR values during the period 2017–2021. The purple color illustrates the number of products and THCL uptake for total products; the blue color illustrates the number of products and THCL uptake for eligible products.

Figure 5 describes the uptake of the THCL logo for total products and eligible products distributed according to their HSR values during the period between 2017 and 2021. Overall, the healthfulness of eligible products varied in a wide range from 0.5 to 5 stars, with the proportion of total products found to be positively correlated with the healthfulness level (black dashed line). Nearly half of the products scoring HSR ≥ 3.5 (855/1862 products, 45.9%) were eligible for bearing THCL, with the highest proportion found among products scoring HSR 5.0 (113/168 products, 67.3%). Notably, the findings also revealed that one-fifth (1240/5905 products, 21.0%) of products scoring HSR ≤ 3.0 were also eligible for labeling with THCL. Exceptionally, this proportion was also found among products scoring HSR 0.5 (129/1213 products, 10.6%).

When looking at the THCL uptake for total products, products receiving a higher HSR (meaning ‘healthier’) were more likely to register to bear the THCL logo (purple line). In particular, products scoring HSR 5.0 had the highest proportional uptake, with 30.5%, followed by products scoring HSR 4.5 (15.8%), HSR 3.5 (13.6%), and HSR 4.0 (10.8%). On the other side, the lowest uptake of the THCL logo was observed in ‘less healthy’ products scoring HSR 0.5 (4.6%) and HSR 1.0 (5.4%).

Paradoxically, this trend of uptake was not found in eligible products (blue line). Specifically, eligible products scoring at a lower tier of HSR (HSR ≤ 2.5) were more likely to register to display the THCL logo, while products scoring at a higher tier of HSR 3.0–4.5 (except HSR 5.0) had a significantly lower rate of registering to bear THCL (the average percentage was 31.0% vs. 44.8%, *p* < 0.001).

**Figure 6:**
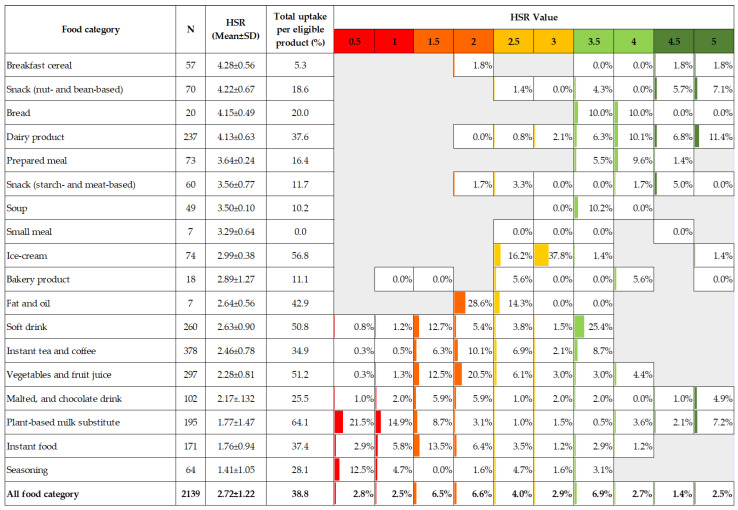
Uptake of THCL as a percentage of eligible products and their corresponding HSR distribution. Note: Values indicate the actual percentage uptake of THCL by their HSR values. Color coding from dark green to dark red indicates the healthfulness grading, classified based on the HSR value (dark red: HSR 0.5–1.0; orange: HSR 1.5–2.0; yellow: HSR 2.5–3.0; light green: HSR 3.5–4.0; dark green: HSR 4.5–5.0). Abbreviations: HSR, Health Star Rating; SD, Standard Deviation.

In order to gain a deeper understanding of the paradoxes surrounding the uptake of THCL, we conducted a detailed analysis by categorizing eligible products based on their healthfulness levels and specific food categories (Figure 6). The findings reveal that products with an HSR score equal to or less than 2.0 exhibited the highest uptake in the seasoning, fat, and oil product category, as well as the instant food and sugar-sweetened beverage groups. On the other hand, products scoring between 2.5 and 3.0 had the highest uptake in the ice cream category. Products with HSR scores of 3.5 or higher were primarily observed in the breakfast cereal, nuts, bread, dairy products, and prepared meals and soups categories.

### 3.4. THCL Uptake by Manufacturer

During the period between 2017 and 2021, newly launched products were reported by 1875 manufacturers. Among them, only 192 (10.2%) manufacturers launched ‘healthier’ products that display THCL logos.

Table 2 provides individual results for manufacturers with ≥5 THCL-labeled products. In general, food manufacturers’ uptake of THCL also varied. Manufacturers with the highest proportionate uptake across their portfolios were beverage companies (MN Beverage, 4Care, Green Spot, Sermsuk, etc.), snack companies (Mae-Ruay Snack Food Factory), and frozen dessert companies (Perfect Companion Group), though there was variation in the number of products made by these manufacturers.

Among larger multinational manufacturers, some companies had an uptake of >70%, such as the Coca-Cola Company (95.7%), Suntory (90.5%), CP-Meji (87.5%), Oishi Group (76.2%), Dutch Mill (73.9%), and Ajinomoto (70.6%), while others had less than 70%, such as PepsiCo (67.9%), Unilever (66.1%), and Nestlé (47.1%). At the bottom of the table, Ichitan, Charoen Pokphand, and Friesland Campina showed the lowest uptake, with proportions of 24.1%, 27.5%, and 29.2%, respectively. Notably, from this analysis, we also found that the uptake by large retailer chains (i.e., supermarkets and convenience stores) was relatively low, such as Tesco (28.6%), Central Retails (25.0%), and Seven iHoldings (10.0%) (data are omitted).

## 4. Discussion

Five years since its adoption commenced, the voluntary uptake of THCL in Thailand has been increasing but seems to remain modest and uneven, which may limit its public health impact. In this investigation, the overall uptake of THCL across newly launched products in packaged food and beverage categories 5 years after implementation was found to be 10.7%. However, the uptake for eligible products was up to 39.5%. Instant foods and non-alcoholic beverages have garnered significant support from food manufacturers, with uptake exceeding 20% of total products and 40% of eligible products. Specifically, sugar-sweetened beverages exhibited uptake rates of 25.3% and 46.0%, respectively. Notably, a thorough analysis of the trendline revealed a progressive increase in the uptake of the Thailand Healthier Choice logo (THCL) over time within these food categories. This encouraging trend can be attributed to a combination of factors, including heightened consumer awareness, improved regulatory measures, and the expansion of public health campaigns [18]. Previous studies have examined the adoption of voluntary front-of-pack nutrition labeling (FOPNL) schemes in various countries, analyzing the accumulation of labeled products on the market. A recent study focused on the Healthier Choice Singapore (HCS) scheme, a similar FOPNL program to THCL, which was reintroduced in September 2015. The findings revealed that after 6 years of implementation, HCS products accounted for approximately 29% of the retail market share. Currently, there are more than 4000 products across 100 food categories displaying one of the 30 HCS logos [19].

HSR, a summary, graded, and monochrome FOPNL scheme, was adopted in NZ and Australia. A study conducted in New Zealand in 2016, 2 years after the adoption of HSR, reported that 5.3% of packaged products surveyed displayed HSR labels [20]. Meanwhile, 10% of products displayed Nutri-Score, a graded and color-coded FOPNL system, on the FOP after one year of implementation in Belgium [21]. In addition, 5 years after the adoption of the HSR in Australia, the uptake was found at 40.7% of eligible food products and beverages [11]. On the other hand, in a recent investigation of Nutri-Score in France and Belgium, a high uptake was found. About 89% of products using the Nutri-Score were sold in French hypermarkets, supermarkets, and specialized retailers after 3 years of implementation [22]. The differences in the uptake progress might reflect the distinct nature of the form of FOPNL (endorsement vs. graded scheme) and other driving factors, such as regulatory actions, industry initiatives, consumer demand, health advocacy, and research findings [6,23].

By analyzing the uptake and eligibility of THCL by food category and healthfulness level, we found several interesting issues related to implementing THCL in Thailand 5 years after its adoption. Basically, the THCL scheme aims to identify and promote healthier choices within the same food group. Although applied to individual food groups (42 food subcategories in the THCL scheme vs. 6 food groups in HSR), the average HSR score and its distribution among eligible products were relatively aligned with the rigidity of the THCL criteria for bearing this symbol. As evidence, the average HSR of eligible products is significantly higher than the HSR of ineligible products in most (94%) food groups (Supplementary S6). Nevertheless, the criteria among some specific food groups (e.g., sugar-sweetened beverages, seasoning, and instant foods) were uneven and appear to be less strict than those for other groups, which allowed products eligible for bearing THCL to still express a low value of HSR. Evidently, the current THCL’s threshold for total sugar in sugar-sweetened beverages (soft drinks, juice drinks, instant tea and coffee, etc.) at 6.0 g/100 mL, which is considerably higher than those set in the Healthier Choice Singapore (threshold of 5.0 g/100 mL) [24] and International Choices criteria (threshold of 2.5 g/100 mL) [25]. In addition, the sodium benchmarks of the THCL criteria for water-based sauces (e.g., fish sauces and soy sauces) and instant noodles/porridge were, respectively, set from 5000–6000 mg/100 mL and 1000 mg/50 g serving [8], whereas the cut-off points for similar groups in the HCS scheme were 4000 mg/100 mL and 500 mg/100 g [24], respectively, and for the International Choices scheme, the thresholds for sodium among dark sauces and flavored noodles/pasta were set at 3000 mg/100 mL and 500 mg/100 g [25]. Thus, in order to improve the performance of THCL, we suggest that the preset nutrient criteria for some food categories be revised.

Secondly, we noticed that THCL is still only displayed in a minority of eligible product types with distinct points of healthiness levels and food groups, which might reflect the commercial reality of marketing and its financial benefits. On the one hand, products that had high-level uptake of THCL were found to be those that are apparently healthy (HSR = 5.0), such as cereals, yogurt, or baked nuts and legumes. A high uptake of THCL was also consistently found in ‘non-core’ food groups, such as instant foods (e.g., instant noodles and instant porridge) and non-alcoholic beverages (e.g., soft drinks and carbonated drinks). The differential use of the THCL logo on these products is unsurprising given the voluntary nature of the system but confirms the perception that some companies are using THCL primarily as a marketing tool, which may alter consumers’ evaluations and food choices [26]. Conversely, in other “core” food categories such as bread, seasoning, and fats and oils, the adoption of THCL remains notably low. These particular products are unlikely to voluntarily display the THCL logo, as it offers limited marketing advantages. As a result, the selective use of THCL, particularly in “non-core” and low-HSR-score foods, alongside consumers’ misinterpretation of THCL labeling, contributes to marketing benefits for manufacturers. As implementation is voluntary, beyond economic considerations, we also noticed that the uptake of THCL products might also depend on the technological challenges in the reformulation and product development process, which need to be balanced between many factors, such as production costs, the availability of common supplies, safety, and shelf life, along with consumer preferences and demands, government regulation, and company profits [27]. Therefore, results that show an inconsistent THCL uptake distribution suggest that the acceleration of the uptake of THCL will require the increased participation of manufacturers in other food categories with significant challenges in reformulation/development, such as small meals, soup, prepared meals, etc.

Product reformulation to increase the availability of healthier products is one of the effective strategies to address obesity and promote healthier food consumption [28]. Encouraging food companies to voluntarily adopt front-of-package labeling by opening a dialogue with industry stakeholders, demonstrating the benefits of such labeling for consumer trust and brand reputation, and showcasing successful case studies can motivate companies to implement front-of-package labels on their products. Recognizing and rewarding companies that take proactive steps in this regard can also incentivize wider adoption [29,30].

Furthermore, we also suggest that the regulated food category be extended to increase the consistency of the FOPNL scheme. This may help avoid confusion and increase consumer trust and allows consumers to compare products more easily and make informed choices [31]. Currently, THCL’s symbol criteria classify products into two distinct groups: eligible for a logo or not. Research evidence shows that, beyond front-of-pack labeling, other governmental food policies may be coherently set, such as restricting advertising to children, product reformulation, financial incentives and disincentives, school food environment standards, etc. [32,33]. Thus, the expansion of THCL’s regulated food categories would be the basic background, and it also suggests a potential for future research to explore THCL’s capacity with the extension of its logo criteria, which identify not only healthier products in a food group but also the least healthy products (e.g., ‘Nutri-Grade’ or International Choices 5-Level Criteria) [34,35].

Thirdly, our results might provide insights for government policymakers for the next phase of THCL implementation. During the first 5 years after its adoption, we suggest that THCL remain voluntary to allow attention to be focused on improvements in implementation and criteria development and revision. However, clear targets and the allocation of responsibilities should be established, and all stakeholders (e.g., government bodies, food manufacturers and retailers, caterers, and other actors) should work together to drive uptake. If THCL does not perform well, the option of mandatory THCL should be studied and taken into consideration. These findings in the present study provide insight into the feasibility of this target. If a linear trend for uptake is maintained, only a 30% uptake by newly launched products will be theoretically reached by 2027. Therefore, accelerating implementation and encouraging more incentives for the food industry to continue uptake must be taken into consideration. A genuine commitment from the government to increase the level of state supervision, the publication of annual interim targets, and the transparent, regular evaluation and monitoring of year-on-year progress would support accountability toward this goal [36].

In this investigation, during the 5 years of implementation between 2017 and 2021, we found that 192 manufacturers launched products displaying THCL. The uptake of THCL varied regardless of the size, type of manufactured food, and type of company. By analyzing the list of companies, our results show that there is still room for increasing the uptake of THCL among small and medium enterprises (SMEs). Providing technical support and capacity building is crucial for their growth, competitiveness, and sustainability. Moreover, supermarkets and convenience store chains can also actively promote and support the uptake of THCL by increasing the labeled products in their private-label product portfolios, empowering consumers to make healthier food choices (e.g., store layout and organization, incentives and promotions), and fostering a culture of transparency and informed decision making within their stores. As the eligible food groups are continuously expanding annually (i.e., 8 food groups in 2017 to 14 food groups in 2023), the expansion and encouragement of the participation of other food manufacturers are possible. This suggests that continued uptake will require constructive and strategic engagement to receive buy-in from a range of remaining manufacturers. Although conducted as a quantitative analysis, our findings also suggest that there are different reasons for transnational corporations and smaller manufacturers not to participate in the THCL scheme. Further qualitative studies should be conducted in order to understand the barriers and motivations of these companies.

In the present report, some limitations should be acknowledged. Firstly, this analysis only covered newly launched packaged foods and beverages in the Thai market, which might not reflect the retention of products on the market and the absolute coverage of all available products nationwide. However, this dataset is robust for time trends. THCL uptake for 2016 was estimated as zero, given the absence of the systematic collection of THCL data at this point in time, and there were likely a small number of products displaying THCL logos by the end of 2016. Secondly, it is possible that the presence of THCL logos on some food labels may have been missed at the time of data collection, as some endorsed products take some time to be re-labeled, and this could also lead to an under-estimation of the uptake of THCL labeling. Thirdly, it is acknowledged that information on FVNL and fiber is not currently mandatory on the standard back-of-pack nutrition information panel in Thailand, and therefore, these values were estimated from ingredient lists, food composition databases, and other sources. It is also worth recognizing that the HSR algorithm is still currently under review and may be subject to updates that could impact HSR values received by products in the future.

## 5. Conclusions

The initial five-year implementation of the THCL program shows promise, but its progress is moderate and inconsistent, which hinders its potential as an effective public health intervention against obesity and NCDs. The findings from this study illustrate the limits of commercial goodwill in applying FOPNL voluntarily. To ensure ongoing effectiveness, it is necessary to establish clear targets and timelines for uptake, along with transparent and regular monitoring. The possibility of making the THCL scheme mandatory should be considered to maximize its impact on public health, providing consumers with a genuine tool for making informed food choices and promoting healthier eating habits.

## Figures and Tables

**Table 1:** Uptake of THCL by food category among total newly launched products and eligible products.

Food Category	Products Displaying THCL/ Total Products *		Products Displaying THCL/Total Eligible Products **		Eligible Products/ Total Products		Linear Trend of Annual Uptake for Total Products ^#^		Linear Trend of Annual Uptake for Eligible Products ^#^	
	n/N	%	n/N	%	n/N	%	%	R^2^	%	R^2^
Bakery products ^a^	2/600	0.3	2/11	18.1	11/600	1.8	0.0	0.0171	0.7	0.0171
Bread ^a^	4/124	3.2	4/11	36.4	11/124	8.9	0.9	0.8359	10.3	0.6171
Breakfast cereal ^a^	3/141	2.1	3/31	9.7	31/141	22.0	0.8	0.6627	4.3	0.6187
Dairy products ^b^	89/532	16.7	89/237	37.6	237/532	44.5	4.8	0.7326	9.9	0.757
Fats and oils ^b^	3/48	6.3	3/7	42.9	7/48	14.6	5.5	0.7024	15.7	0.4939
Ice cream ^b^	42/317	13.2	42/74	56.8	74/317	23.3	4.4	0.9402	15.8	0.8909
Instant foods ^b^	64/266	24.1	64/171	37.4	171/266	64.3	9.7	0.9203	13.3	0.9312
Instant tea and coffee ^b^	132/559	23.6	132/378	34.9	378/559	67.6	7.8	0.9707	10.8	0.9773
Malted and chocolate drinks ^b^	26/149	17.4	26/102	25.5	102/149	68.5	6.5	0.8723	8.0	0.7936
Plant-based milk substitutes ^b^	125/332	37.7	125/195	64.1	195/332	57.8	8.6	0.6642	14.6	0.7834
Prepared meals ^b^	12/534	2.2	12/73	16.4	73/534	13.7	0.6	0.1098	4.3	0.1813
Seasoning ^b^	18/322	5.6	18/64	28.1	64/322	19.9	2.3	0.7215	9.2	0.8028
Small meals ^b^	0/84	0.0	0/7	0.0	7/84	8.3	N/A	N/A	N/A	N/A
Snacks (starch- and meat-based) ^b^	7/2112	0.3	7/60	11.7	60/2112	2.8	0.1	0.5714	3.2	0.3811
Snacks (nut- and bean-based) ^b^	13/359	3.6	13/70	18.6	70/359	19.5	0.4	0.1054	1.6	0.0593
Soups ^b^	5/84	6.0	5/49	10.2	49/84	58.3	1.1	0.0765	1.9	0.115
Soft drinks ^b^	132/512	25.8	132/260	50.8	260/512	50.8	7.1	0.8276	8.7	0.3644
Vegetable and fruit juices ^b^	152/692	22.0	152/297	51.2	297/692	42.9	6.6	0.8947	13.2	0.8704
Total	829/7767	10.7	829/2097	39.5	2097/7767	27.0	3.0	0.9289	10.2	0.8761

* Total products are total newly launched products, either with or without displaying Healthier Choice nutrition labeling. ** Total eligible products are total newly launched products that passed the Thailand Healthier Choice scheme’s criteria, either with or without displaying Healthier Choice nutrition labeling. ^#^ The linear trendline was estimated, and the slope equation is shown in Supplementary S5. ^a^ Number of total products and total eligible products accumulated between 2019 and 2021. ^b^ Number of total products and total eligible products accumulated between 2017 and 2021. Abbreviations: THCL, Thailand Healthier Choice nutrition labeling; N/A, Not Available.

**Table 2:** THCL uptake among total eligible products, classified by food manufacturer.

Company *	Products Surveyed (N)	Products with THCL Labeling/ Products Eligible for THCL Labeling (n/N)	THCL Labeling (%)
MN Beverage	29	26/26	100.0
4Care	8	6/6	100.0
Green Spot	32	13/13	100.0
Sermsuk	26	7/7	100.0
Mae-Ruay Snack Food Factory	38	7/7	100.0
Perfect Companion Group	34	6/6	100.0
The Coca-Cola Company	52	22/23	95.7
Dairy Plus	33	14/15	93.3
Suntory	40	19/21	90.5
CP-Meiji	41	14/16	87.5
Taveephol Product	28	7/8	87.5
Uni-President Enterprises	28	12/14	85.7
Nam-Chao	25	6/7	85.7
Tofusan	49	35/41	85.4
Asahi Group	17	8/10	80.0
Medifoods	9	7/9	77.8
Oishi Group	40	16/21	76.2
Kewpie	16	6/8	75.0
Dutch Mill	48	17/23	73.9
Toyo Seikan	11	8/11	72.7
Ajinomoto	34	12/17	70.6
PepsiCo	247	38/56	67.9
Malee Group	48	10/15	66.7
Meiji	55	10/15	66.7
Unilever	145	37/56	66.1
Fraser and Neave	22	7/11	63.6
Doi Kham Food Products	39	10/16	62.5
Chabaa Bangkok	32	8/13	61.5
T.C. Pharmaceutical Industries	64	14/26	53.8
Thai President Foods	61	12/25	48.0
Nestlé	161	49/104	47.1
Nissin Foods	35	7/18	38.9
Sappe	47	11/32	34.4
FrieslandCampina	66	7/24	29.2
Tesco	144	10/35	28.6
Charoen Pokphand	224	11/40	27.5
Ichitan Group	51	7/29	24.1
All other companies	5688	313/1668	18.8
Total	7767	829/2097	39.5

* Results are extracted from Mintel Global New Products Database and are listed individually for manufacturers with ≥5 products displaying the THCL logo.

## Data Availability

Not applicable.

## Supplementary Materials

The following supporting information can be downloaded at https://www.mdpi.com/article/10.3390/nu15143116/s1: Supplementary S1: Thailand Healthier Choice nutrition labeling criterion and its food categories and subcategories; Supplementary S2: Timeline of Thailand Healthier Choice nutritional labelling Food Category Expansion; Figure S1: Timeline of Thailand Healthier Choice nutrition labeling food category expansion; Supplementary S3: Food categories and subcategories in Mintel Global New Product Database; Supplementary S4: Food categories used in the present study and their definitions; Supplementary S5: Linear equation and R-squared value of uptake of Thailand Healthier Choice nutrition labeling for total newly launched products and eligible products; Supplementary S6: Mean ± standard error of Health Star Rating values of eligible and ineligible products for bearing Thailand Healthier Choice nutrition labeling by food category.
