# Supplementary material for: Uptake of Front-of-Package Nutrition Labeling Scheme after 5 Years of Adoption in Thailand: An Analysis of New Launched Pre-Packaged Food and Beverages Products
Source: Nutrients. 2023 Jul 12;15(14):3116. doi: 10.3390/nu15143116 (PMC10385136; doi:10.3390/nu15143116)
Supplement: Supplementary file 1 [file nutrients-15-03116-s001.zip › nutrients-2466067-supplementary.pdf]

Supplementary S1: Thailand Healthier Choice Nutritional Labelling Criterion and Its Food Category and Subcategory (update in April 2023 - 14 food categories)

Food category		Food sub-category	Nutritional criteria	
1. NON-ALCOHOLIC BEVERAGES	1.	**Fruit juice, vegetable juice 100%**	Total Sugar	≤ 12 gr per 100 ml and<br>≤ 18 gr per package
			Sodium	≤ 100 mg per 100 ml
			Dietary fiber	≥ 0.75 gr per 100 ml
	2.	**Fruit juices/ Vegetable juices, soft drinks, and sweetened beverages with various flavors**	Total Sugar	≤ 6 gr per 100 ml and<br>≤ 18 gr per package. One serving is not more than 150% of the reference serving size and the package size is not more than 500 ml.
			Sodium	≤ 40 mg per 100 ml<br>≤100 mg per 100 ml, apply with tomato juice that is greater than or equal to 90%.
			Total Energy	≤ 40 kcal per 100 ml<br>≤ 24 kcal per 100 ml, in case the ingredient contains sugar and sugar substitutes<br>≤ 10 kcal per 100 ml, in case the ingredient contains only sugar substitutes
	3.	**Cereal milk, soy milk**	Total sugar	≤ 6 gr per 100 ml and<br>≤ 18 gr per package. One serving is not more than 150% of the reference serving size and the package size is not more than 500 ml.
			Total fat	≤ 3.5 gr per 100 ml
			Saturated fat	≤ 1 gr per 100 ml
			Sodium	≤ 40 mg per 100 ml
	4.	**Chocolate beverages, cocoa, malt extracts, and constituents of milk or protein**	Total Sugar	≤ 6 gr per 100 ml and<br>≤ 18 gr per package. One serving is not more than 150% of the reference serving size and the package size is not more than 500<br>ml.
			Total Fat	≤ 1 gr per 100 ml and<br>≤ 3 gr per package. One serving is not more than 150% of the reference serving size
			Sodium	≤ 100 mg per 100 ml
			Total energy	≤ 60 kcal per 100 ml<br>≤ 40 kcal per 100 ml, in case the ingredient contains sugar and sugar substitutes
	5.	**Instant tea**	Total Sugar	≤ 6 gr per 100 ml and<br>≤ 18 gr per package. One serving is not more than 150% of the reference serving size and the package size is not more than 500 ml.
			Total Fat	≤ 0.6 gram per 100 ml and<br>≤ 1.8 grams per package. One serving is not more than 150% of the reference serving size
			Sodium	≤ 40 mg per 100 ml
			Total energy	≤ 40 kcal per 100 ml<br>≤ 24 kcal per 100 ml, in case the ingredient contains sugar and sugar substitutes<br>≤ 10 kcal per 100 ml, in case the ingredient contains only sugar substitutes
	6.	**Instant coffee**	Total Sugar	≤ 6 gr per 100 ml and<br>≤18 gr per package. One serving is not more than 150% of the reference serving size and the package size is not more than 500 ml.

		Total Fat	≤ 1 gr per 100 ml and<br>≤ 3 gr per package. One serving is not more than 150% of the reference serving size
		Sodium	≤ 100 mg per 100 ml
		Total energy	≤ 60 kcal per 100 ml<br>≤ 40 kcal per 100 ml, in case the ingredient contains sugar and sugar substitutes
2. SEASONING SAUCES	**7. Fish sauce**	Sodium	≤ 6,000 mg per 100 ml.
	**8. Seasoning, soy sauce**	Sodium	≤ 5,000 mg per 100 ml.
	**9. Black soy sauce**	Sodium	≤ 5,000 mg per 100 ml.
	**10. Sweet soy sauce**	Sodium	≤ 2,000 mg per 100 gr.
		Total sugar	≤ 40 g per 100 gr
	**11. Other Sweet sauce**	Sodium	≤ 900 mg per 100 gr
		Total sugar	≤ 30 gr per 100 gr
	**12. Oyster sauce**	Sodium	≤ 2,000 mg per 100 gr
		Total sugar	≤ 11 gr per 100 gr
	**13. Chili sauce**	Sodium	≤ 750 mg per 100 gr
		Total sugar	≤ 12 gr per 100 gr
	**14. Tomato sauce**	Sodium	≤ 750 mg per 100 gr
		Total sugar	≤ 17 gr per 100 gr
	**15. Other sauces for dipping**	Sodium	≤ 1,300 mg per 100 gr
		Total sugar	≤ 14 gr per 100 gr
	**16. Other Western style sauces**	Sodium	≤ 800 mg per 100 gr
		Total sugar	≤ 16 gr per 100 gr
	**17. Suki sauce**	Sodium	≤ 1,500 mg per 100 gr
		Total sugar	≤ 15 gr per 100 gr
3. DAIRY PRODUCTS	**18. Fresh milk, powdered milk**	Total Sugar	No added sugar
		Total fat	≤ 4 gr per 100 ml, except if one serving contains more than 150% of the reference serving size, must not have added sugar and total fat not more than 12 grams.
	**19. Flavored Milk Product**	Total Sugar	≤ 8 gr per 100 ml, except if one serving contains more than 150% of the reference serving size, total sugar must not exceed 24 gr.
	**20. Cultured Milk (regular yoghurt, yoghurt drinks)**	Total fat	≤ 3.5 g per 100 ml, except if one serving contains more than 150% of the reference serving size, total fat must not more than 10.5 g.<br>Notes: *One reference serving size of milk and ready-to-drink milk products is 200 ml. 150% of one reference serving size of milk and dairy products or RTD milk equal to 300 ml.*
		Total Sugar	≤ 5 gr per 100 ml, except if one serving contains more than 150% of the reference serving size, total sugar must not exceed<br>11.25 g.<br>Notes: *One reference serving size of milk and ready-to-drink milk products is 150 ml. 150% of one reference serving size of semi-liquid cultured milk, semi-solid cultured milk and ready-to-drink cultured milk equal to 225 ml.*
	**21. Powdered milk products**		Consider giving nutrition symbols only to recipes that have a brewing method with the addition of water only.
4. INSTANT FOOD	**22. Instant noodle**	Sodium	≤ 1,000 milligrams per 50 grams for packaging with no more than 70 g per serving, except if one serving is more than 70 g, shall contain no more than 1,400 mg of sodium per serving.
	**23. Rice porridge or congee**	Sodium	≤ 1,000 milligrams per 50 grams for packaging with no more than 50 g per serving, except if one serving is more than 50 g, shall contain no more than 1,000 mg of sodium per serving.
		Total fat	≤ 4 g per 100 g prepared product

	24.	**Plain soup and concentrate soup (cube, powder, dried)**	Sodium	≤ 240 mg per 100 g prepared product<br>Note: This criterion means the soup should be in powdered or cubed form and added with water for immediate consumption, the product shall does not include soup cubes used as raw materials for cooking
**5. SNACK**	25.	**Snacks not included nuts and beans (baked, fried, salted or flavored)**	Total energy<br>Saturated fat<br>Total sugar<br>Sodium	≤ 150 kcal per serving<br>≤ 6 gr per 100 gr<br>≤ 7 gr per 100 gr<br>≤ 500 mg per100 gr
	26.	**Beans, nuts and seed**	Total energy<br>Total fat<br>Total Sugar<br>Sodium	≤ 200 kcal/Serving<br>≤ 0.3% (no added oiled, except to enhance flavor)<br>No added sugar<br>≤ 100 mg per 100 gr
**6. ICE CREAM**	27.	**Ice cream all kinds**	Total energy<br>Saturated fat<br>Total Sugar<br>Sodium	≤ 130 kcal per serving<br>≤ 5 gr per 100 gr<br>≤ 20 gr per 100 gr<br>≤ 100 mg per 100 gr
**7. FAT AND OIL**	28.	**Margarine**	Saturated fat<br>Sodium	≤ 27gr per 100 gr<br>≤ 100 mg per100 gr
	29.	**Mayonnaise, Salad dressing and Sandwich spread**	Total energy<br>Saturated fat<br>Total Sugar<br>Sodium	≤ 350 kcal per 100 gr<br>≤ 4 gr per 100 gr<br>≤ 15 gr per 100 gr<br>≤ 750 mg per 100 gr
**8. BREAD**	30.	**Bread without loaves**	Total sugar<br>Total fat<br>Sodium<br>Dietary fiber	≤ 6 gr per 100 gr<br>≤ 5 gr per 100 gr<br>≤ 450 gr per 100 gr<br>≥ 3 mg per 100 gr
**9. BREAKFAST CEREAL**	31.	**Breakfast Cereal**	Total sugar<br>Saturated fat<br>Sodium<br>Dietary fiber	≤ 20 gr per 100 gr<br>≤ 3 gr per 100 gr<br>≤ 300 gr per 100 gr<br>≥ 6 mg per 100 gr
**10. BAKERY PRODUCTS**	32.	**Cookies and cakes all kinds**	Total energy<br><br>Total sugar<br>Total fat<br>Sodium<br>Dietary fiber	≤ 110 kcal per serving. Reference and one serving shown on the label.<br>≤ 20 gr per 100 gr<br>≤ 22 gr per 100 gr<br>≤ 300 gr per 100 gr<br>≥ 3 mg per 100 gr
**11. SMALL MEAL**	33.	**Salty/meat fillings such as sandwiches, hamburgers, stuffed buns and buns**	Total energy<br>Total sugar<br>Total fat<br>Sodium	≤ 150-250 kcal per serving<br>≤ 2.5 gr per 100 kcal<br>≤ 3.25 gr per 100 kcal<br>≤ 175 mg per 100 kcal
	34.	**Sweet filling/cream filling/custard/jam/fruit such as sandwiches, stuffed bread and buns**	Total energy<br>Total sugar<br>Total fat<br>Sodium	≤ 150-250 kcal per serving<br>≤ 3.75 gr per 100 gr<br>≤ 3.25 gr per 100 gr<br>≤ 150 mg per 100 gr
**12. FISH AND OTHER AQUATIC PRODUCTS**	35.	**Fish fillets in mineral water and brine**	Total Sugar<br>Total Fat<br>Sodium	No added sugar<br>No added oil<br>≤ 350 mg per 100 gr
	36.	**Fish fillets in oil**	Total Sugar<br>Total fat<br>Sodium	No added sugar<br>≤ 5 gr per 100 gr<br>≤ 350 mg per 100 gr
	37.	**Fish fillet in tomato and chili sauce**	Total sugar<br>Total fat<br>Sodium<br>Total sugar	≤ 2 gr per 100 gr<br>≤ 5 gr per 100 gr<br>≤ 400 mg per 100 gr<br>≤ 5 gr per 100 gr

13. MEAT PRODUCTS	38.	**All kinds of sausages, meat ball (including Vietnamese sausages, chicken ball etc.)**	Total Sugar	2.5 gr per 100 gr
			Sodium	≤ 550 mg per 100 gr (Phase 1)
			Total Fat	≤ 10 gr per 100 gr or 10-15 gr per 100 gr if the proportion of saturated fatty acids must be 45% of the total fat
	39.	**Ham**	Total Sugar	2.5 gr per 100 gr
			Sodium	≤ 900 mg per 100 gr
			Total Fat	≤ 10 gr per 100 gr
	40.	**Seasoned meat**	Total Sugar	2.0 gr per 100 gr
			Sodium	≤ 450 mg per 100 gr
			Total Fat	≤ 8 gr per 100 gr
	41.	**Meat slices/ sticks/ crispy, meat shredded**	Total Sugar	25 gr per 100 gr
			Sodium	≤ 1,000 mg per 100 gr
			Total Fat	≤ 8 gr per 100 gr

14. PREPARED MEAL: Ready-to-eat meal includes food that is consumed as a main dish of complete nutrients especially calories, carbohydrate, protein, and fat. Ready-to-eat meals in both frozen and chilled forms usually provide 250-500 kcal per serving. Qualified food products in this category must meet all following criteria: (1) Having energy between 250-500 kilocalories per serving; (2) Fat, saturated fat, sugar, and sodium score must be higher than 0; (3) Total scores must be 20 or more out of 40. Scores of nutrients per 100 kcal are shown in the below table.

Type of nutrient	Unit	Score					
		0	1	2	3	4	5
**Protein**	g	< 0.50, > 25*	0.50 – 1.00	1.01 – 1.50	1.51 – 2.00	2.01 – 2.50	> 2.50, ≤ 25*
**Calcium**	mg	< 8, > 750*	8 – 16	17 – 24	25 – 32	33 – 40	> 40, ≤ 750*
**Iron**	mg	< 0.14, > 11.25*	0.14 – 0.28	0.29 – 0.42	0.43 – 0.56	0.57 – 0.70	> 0.70, ≤ 11.25*
**Fiber**	g	< 0.25	0.25 – 0.50	0.51 – 0.75	0.76 – 1.00	1.01 – 1.25	> 1.25
**Total Fat**	g	> 3.25	2.94 – 3.25	2.62 – 2.93	2.30 – 2.61	1.98 – 2.29	≤ 1.97
**Saturated Fat**	g	> 1.00	0.96 – 1.00	0.91 – 0.95	0.86 – 0.90	0.81 – 0.85	≤ 0.80
**Total Sugar**	g	> 1.25	1.01 – 1.25	0.76 – 1.00	0.51 – 0.75	0.25 – 0.50	< 0.25
**Sodium (Phase 1)**	mg	< 75, > 175	156 – 175	136 – 155	116 – 135	96 – 115	75 - 95

* Amount per one serving

Supplementary S2: Timeline of Thailand Healthier Choice nutritional labelling Food Category Expansion (update in April 2023 - 14 food categories)

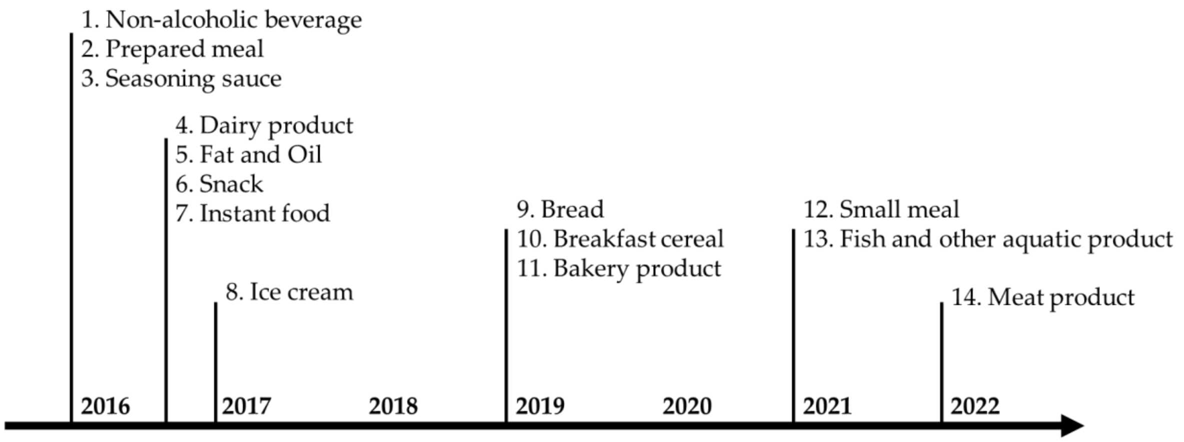

Figure S1. Timeline of THCL Food Category Expansion

Supplementary S3: Food category and subcategory in Mintel Global New Product Database (GNPD)

Food category		Sub-category	
1.	Bakery products	1.	Sweet Biscuits/Cookies
		2.	Bread & Bread Products
		3.	Savory Biscuits/Crackers
2.	Breakfast cereals	4.	Cold Cereals
		5.	Hot Cereals
3.	Carbonated soft drinks	6.	Carbonated soft drinks
4.	Dairy	7.	Plant Based Drinks (Dairy Alternatives)
		8.	Flavored Milk
		9.	White Milk
		10.	Drinking Yogurt & Liquid Cultured Milk
5.	Desserts & Ice Cream	11.	Dairy-Based Ice Cream & Frozen Yogurt
		12.	Plant Based Ice Cream & Frozen Yogurt (Dairy Alternatives)
		13.	Water Based Ice Lollies, Pops & Sorbets
6.	Hot Beverages	14.	Malt & Other Hot Beverages
		15.	Coffee
		16.	Tea
7.	Juice Drinks	17.	Nectars
		18.	Fruit/Flavoured Still Drinks
		19.	Juice
8.	Meal and Meal Centers	20.	Prepared Meals
		21.	Instant Noodles
		22.	Instant Rice
9.	Nutritional Drinks & Other Beverages	23.	Nutritional & Meal Replacement Drinks
10.	RTDs (Ready to Drinks)	24.	RTD (Iced) Tea
		25.	RTD (Iced) Coffee
11.	Sauces & Seasonings	26.	Table Sauces
		27.	Dressings & Vinegar
12.	Snacks	28.	Bean-Based Snacks
		29.	Rice Snacks
		30.	Fruit Snacks
		31.	Potato Snacks
		32.	Meat Snacks
		33.	Popcorn
		34.	Corn-Based Snacks
		35.	Vegetable Snacks
		36.	Nuts
		37.	Cassava & Other Root-Based Snacks
		38.	Snack Mixes
		39.	Snack/Cereal/Energy Bars
		40.	Wheat & Other Grain-Based Snacks
13.	Soup	41.	Wet Soup
14.	Sports and Energy Drinks	42.	Energy Drinks
		43.	Sports Drinks
15.	Water	44.	Flavored Water
		45.	Water

Supplementary S4: Food category using in the present study and its definition

No.	Food Category	Definition
**1**	Bakery products	Bakery products, which include rolls, cookies, pies, pastries, and muffins, except bread which are usually prepared from flour or meal derived from some form of grain.
**2**	Bread	Bread is a kind of food made of flour or meal that has been mixed with milk or water, made into a dough or batter, with or without yeast or other leavening agent, and baked. This category includes bread without loaves.
**3**	Breakfast cereal	Food made from processed grains, such as maize, oats, wheat or corn, rice, is usually eaten for breakfast with milk and sometimes sugar. This category includes both hot and cold cereals such as muesli, granola, flakes etc.
**4**	Dairy product	Dairy products are defined as milk and any of the foods made from milk, including fresh milk, yogurt, condensed and dried milk. This category excludes other dairy-based food products such as butter, cheese, ice cream
**5**	Fats and oil	This category includes dressing, which is a type of sauce prepared for salads that usually has a vinegar and oil base with the addition of herbs and spices
**6**	Ice-cream	Ice-cream is a mixture of dairy ingredients (e.g. milk, cream, nonfat milk), ingredients for sweetening and flavoring, that has been frozen into a soft, creamy delight. Water-based ice lollies, pops & sorbets are included in this category.
**7**	Instant food	Are convenience foods which require minimal preparation, typically just adding water or milk. This food category includes instant noodle and instant porridge
**8**	Instant tea and coffee	Coffee and tea, where milk is not the primary ingredient. May or may not be carbonated. Include powdered, ground, bags, or ready-to-drink coffee/ tea.
**9**	Malted, and chocolate drink	Beverages which contain chocolate and/or cocoa as the main and/or predominant ingredient and/or flavor. Include powdered, ground, or ready-to-drink malted beverages (hereafter referred to as chocolate malted drinks)
**10**	Plant-based milk substitute	Non-dairy beverages consist primarily of liquid extracted from plants for flavoring and aroma. Include grains (cereal, rice), legumes (peas, soy), and other varieties of seeds and nuts.
**11**	Prepared meal	Are defined as pre-prepared or ready-to-eat main courses, supplied in the container under frozen or chilled form, requiring no further ingredients or preparation required other than heating
**12**	Seasoning/ Sauces	Substances are added to other foods to enhance their flavor and smell. This category includes all kinds of sauces such as dark sauces, meal sauces, emulsified sauces, other sauces (water-based) etc.
**13**	Small meal	Are defined as pre-prepared or ready-to-eat light meals such as hors d'oeuvres or canapes. This food category includes both salty/meat fillings such as sandwiches, hamburgers, stuffed buns and sweet /cream filling such as sandwiches, stuffed bread and buns etc.
**14**	Snack (starch- and meat-based)	Are a small amount of food that is eaten between meals, or a very small meal, formed under packaged and/or processed foods, which include tortilla chips, corn chips, potato chips, extruded products (direct expansion and pellets), pork rinds, pretzels, popcorn etc.
**15**	Snacks (nut- and bean-based)	Are a small amount of food that is eaten between meals, or a very small meal, formed under packaged and/or processed foods, which include legumes, nut, seed and bean.
**16**	Soups	Soups consist of liquid soups, and/or canned soups. It can be partially dehydrated, or condensed, containing some liquid content.
**17**	Soft drink	Non-alcoholic beverages include carbonated drinks, energy drink, flavored water, sport drinks.
**18**	Vegetables and fruit juice	All fruit and vegetable juice that includes both non-100% and 100% fruit and vegetable juice.

Supplementary S5: Linear equation and R-squared value of uptake of Thailand Healthier Choice nutrition labelling per total newly launched product and eligible product

Food category	Trend of annual uptake per total product*	Trend of annual uptake per eligible product**
Bakery products	y = 0.0246x + 0.0575; R^2 = 0.0171	y = 0.7143x + 1.6667; R^2 = 0.0171
Bread	y = 0.8963x - 1.5585; R^2 = 0.8359	y = 10.286x – 16; R^2 = 0.6171
Breakfast cereal	y = 0.7472x - 1.5095; R^2 = 0.6627	y = 4.2857x - 8.5185; R^2 = 0.6187
Dairy products	y = 4.8297x - 2.5405; R^2 = 0.7326	y = 9.9002x - 4.2153; R^2 = 0.757
Fats and oils	y = 5.4762x - 11.296; R^2 = 0.7024	y = 15.714x – 30; R^2 = 0.4939
Ice-cream	y = 4.3809x - 5.9176; R^2 = 0.9402	y = 15.807x - 15.786; R^2 = 0.8909
Instant food	y = 9.6739x - 13.138; R^2 = 0.9203	y = 13.302x - 15.927; R^2 = 0.9312
Instant tea and coffee	y = 7.8262x - 10.022; R^2 = 0.9707	y = 10.841x - 12.695; R^2 = 0.9773
Malted, and chocolate drink	y = 6.5442x - 7.7831; R^2 = 0.8723	y = 8.0213x - 7.6628; R^2 = 0.7936
Plant-based milk substitute	y = 8.6205x + 1.4308; R^2 = 0.6642	y = 14.598x + 1.81; R^2 = 0.7834
Prepared meal	y = 0.6048x + 0.1363; R^2 = 0.1098	y = 4.2606x - 0.4723; R^2 = 0.1813
Seasoning	y = 2.3005x - 3.1701; R^2 = 0.7215	y = 9.2262x - 12.5; R^2 = 0.8028
Snacks (starch- and meat-based)	y = 0.1217x - 0.139; R^2 = 0.5714	y = 3.2381x - 0.7778; R^2 = 0.3811
Snacks (nut- and bean-based)	y = 0.4467x + 1.488; R^2 = 0.1054	y = 1.5522x + 9.6658; R^2 = 0.0593
Soups	y = 1.0909x + 0.3232; R^2 = 0.0765	y = 1.9388x - 0.2381; R^2 = 0.115
Soft drink	y = 7.122x - 4.2145; R^2 = 0.8276	y = 8.7015x + 11.722; R^2 = 0.3644
Vegetables and fruit juice	y = 6.567x - 4.387; R^2 = 0.8947	y = 13.244x - 4.8496; R^2 = 0.8704

* Total product is total newly launched product, include either product with or without displaying Healthier Choice nutritional labeling;

** Total eligible product is total newly launched product that passed the Thailand Healthier Choice scheme's criteria, and either with or without displaying the Healthier Choice nutritional labeling.

Supplementary S6: Mean ± standard error Health Star rating value of eligible and il-eligible products for bearing Thailand Healthier Choice nutrition labelling, by food category

Food category	Health Star Rating value (Mean ± SE)					
	N	Eligible products	N	Ineligible products	Differences	p-value
Bakery products	11	3.14±0.38	589	1.22±0.03	+ 1.91±0.19	<0.001
Bread	11	4.09±0.15	113	2.81±0.08	+ 1.29±0.27	<0.001
Breakfast cereal	31	4.32±0.11	110	3.07±0.07	+ 1.25±0.15	<0.001
Dairy products	237	4.13±0.04	295	3.40±0.05	+ 0.73±0.07	<0.001
Fats and oils	7	2.64±0.21	41	1.32±0.09	+ 1.33±0.23	<0.001
Ice-cream	74	2.99±0.04	243	2.17±0.04	+ 0.82±0.07	<0.001
Instant food	171	1.76±0.07	95	0.76±0.06	+ 0.99±0.11	<0.001
Instant tea and coffee	378	2.46±0.04	181	0.99±0.05	+ 1.47±0.07	<0.001
Malted, and chocolate drink	102	2.17±0.13	47	0.95±0.16	+ 1.22±0.22	<0.001
Plant-based milk substitute	195	1.77±0.11	137	1.82±0.14	– 0.05±0.17	0.755
Prepared meal	73	3.64±0.03	461	3.29±0.02	+ 0.35±0.48	<0.001
Seasoning	64	1.41±0.13	258	1.04±0.04	+ 0.37±0.11	0.001
Small Meal	7	3.29±0.24	53	2.08±0.12	+ 1.20±0.35	0.001
Snacks (starch- and meat-based)	60	3.56±0.10	2076	1.91±0.02	+ 1.64±0.12	<0.001
Snacks (nut- and bean-based)	70	4.22±0.08	289	3.52±0.05	+ 0.71±0.11	<0.001
Soups	49	3.50±0.01	35	3.27±0.05	+ 0.23±0.05	<0.001
Soft drink	260	2.63±0.06	252	0.82±0.03	+ 1.81±0.07	<0.001
Vegetables and fruit juice	297	2.28±0.05	395	1.39±0.05	+ 0.89±0.07	<0.001
Total	2,097	2.69±0.03	5,670	1.99±0.02	+ 0.71±0.03	<0.001

Values were expressed as mean ± standard error. The difference between HSR value of eligible and ineligible products was compared by Independent-t-test
